# Strategy Changes After Errors Improve Performance

**DOI:** 10.3389/fpsyg.2015.02051

**Published:** 2016-01-12

**Authors:** Liesbet Van der Borght, Charlotte Desmet, Wim Notebaert

**Affiliations:** Department of Experimental Psychology, Ghent UniversityGhent, Belgium

**Keywords:** post-error slowing, post-error accuracy increase, mental arithmetic, strategy-use, cognitive control

## Abstract

The observation that performance does not improve following errors contradicts the traditional view on error monitoring (Fiehler et al., 2005; Núñez Castellar et al., 2010; Notebaert and Verguts, 2011). However, recent findings suggest that typical laboratory tasks provided us with a narrow window on error monitoring (Jentzsch and Dudschig, 2009; Desmet et al., 2012). In this study we investigated strategy-use after errors in a mental arithmetic task. In line with our hypothesis, this more complex task did show increased performance after errors. More specifically, switching to a different strategy after an error resulted in improved performance, while repeating the same strategy resulted in worse performance. These results show that in more ecological valid tasks, post-error behavioral improvement can be observed.

## Introduction

In everyday life, errors come in a lot of forms, which makes it a real challenge to study how humans deal with errors. Giovannetti et al. (2007) investigated participants’ performance during coffee making. Even after practice, participants still made a lot of errors, and they were able to detect most of them. While it is fairly easy to show that people detect errors in everyday life tasks, it is far more complicated to study adaptations after errors in these tasks. The advantage of experimental tasks is that the presentation of hundreds of trials allows the investigation of post-error performance. However, investigating post-error behavior in the lab also has its downsides….

According to cognitive control theories post-error slowing (PES) is the result of an increase in cognitive control (e.g., the conflict monitoring theory: Botvinick et al., 2001). This upregulation of control should lead to a better performance on the next trial, so post-error trials are predicted to be slower and more accurate (Rabbitt, 1979; Brewer and Smith, 1984). This speed-accuracy trade-off has been reported in the literature (e.g., Marco-Pallarés et al., 2008; Danielmeier et al., 2011) but an overview of the literature indicates that this pattern is not reliable. Other studies observed no difference in accuracy after correct trials and errors (Hajcak et al., 2003; Hajcak and Simons, 2008; King et al., 2010) or even decreased accuracy following errors (Fiehler et al., 2005; Núñez Castellar et al., 2010; Notebaert and Verguts, 2011). This gave rise to so-called non-functional explanations for PES. In these accounts, PES is explained as a non-strategic result of an attentional dip following errors (Notebaert et al., 2009) or a result of an error monitoring process occupying a central bottleneck (Jentzsch and Dudschig, 2009). Interestingly, these accounts predict decreased accuracy shortly following an error. Indeed PES seems to decrease with increasing inter-trial intervals (Danielmeier and Ullsperger, 2011; Van der Borght et al., 2015) and longer inter-trial intervals also result in post-error accuracy *increase* (Jentzsch and Dudschig, 2009).

However, the inter-trial interval might not be the only factor influencing post-error adaptation. Previous work from our lab, showed that the task characteristics of the typical paradigms used in cognitive control research might have caused the discrepancy in results concerning post-error adaptations (Desmet et al., 2012). We argued that most tasks used in cognitive control research are very restricted in the adaptation strategies they allow. In most cases, improvements after errors can only be obtained by an update of the stimulus-response rules and a more attentive focus toward the stimulus. As such, more specific types of post-error adjustments, i.e., less errors and faster responses due to selective attention in conflict tasks, have been proposed (Maier et al., 2011). However, whether post-error reduction of interference can indeed be found is still debated (Van der Borght et al., 2014). To test the hypothesis that post-error improvement can be observed in more complex tasks, we turned to a task were multiple solution strategies are at hand, namely a mental arithmetic task. We offered participants multiplication equations and they had to be recognized as true or false. Literature on mental arithmetic has shown that these equations can be solved based of different strategies, for example the correct response (true/false) can be based on familiarity, memory or calculation (Campbell and Fugelsang, 2001; Romero et al., 2006). In our previous study (Desmet et al., 2012), we showed accuracy improvement after errors. In the current experiment, we explicitly ask participants on every trial which strategy they used to solve the arithmetic multiplication. If our hypothesis holds, we should find post-error accuracy increases when participants switch strategies but not when they repeat strategies.

In addition, our design permits us to study the proportion of strategy switches and repetitions after errors. Using a numerosity judgment task, Schillemans et al. (2011) demonstrated that people tend to repeat the previous strategy rather than to switch to another strategy (perseveration effect). Their design was not suited for separating post-correct and post-error trials. With our design we can extent these findings and investigate whether the perseveration effect also holds after errors or not.

## Materials and Methods

### Participants

Twenty students at Ghent University (all females) participated in this study (mean age = 19.7 years, *SD* = 2.6 years). The participants earned course credits in exchange for participation.

### Material

Stimuli were presented on a 17-inch computer screen. The viewing distance was about 50 cm. The multiplication problems were centered on the screen in the traditional format (e.g., 3 × 7 = 21) and presented in white on a black background (total outline: 3.7 cm × 0.6 cm). Responses were recorded by a *Cedrus* response box and a numeric keypad. The experiment was conducted using Tscope software (Stevens et al., 2006).

### Stimuli

The stimuli used are the same as in the experiment of Desmet et al. (2012). Half of the trials comprised problems presented with a correct solution (CORRECT: 4 × 6 = 24). The selected problems ranged from 2 × 3 to 8 × 9. Tie problems were not included (problems with repeated operands, e.g., 3 × 3). Every problem occurred in both the ‘larger × smaller’ and the ‘smaller × larger’ order, resulting in 56 unique problems for correct problem types. These 56 problems were repeated eight times during the experiment. The other half of the trials comprised problems presented with an incorrect solution (distracters). The distracter was always one step away from the correct solution (DISTRACTER: 4 × 6 = 28). We included four different outcomes for each of the 28 distracter problems: (a + 1) × b; (a - 1) × b; a × (b + 1); a × (b - 1). With this set of stimuli the direction of the ‘split’ (i.e., the magnitude difference between the presented distracter and the correct product, Ashcraft and Stazyk, 1981; Koshmider and Ashcraft, 1991) was controlled: half of the distracters were larger than the correct product, the other half was smaller than the correct product. Including the order of larger operand first/smaller operand first, there were 224 distracter problems. Every problem was repeated twice during the experiment. In reality the four different distracter lists sometimes contained the same distracters. This was the case for problems with two or nine as one of the operands (e.g., 2 × 7 or 9 × 3) because problems with one [e.g., (2 - 1) × 7] or 10 as one of the operands [e.g., (9 + 1) × 3] were excluded from the stimulus set.

### Procedure

Participants had to classify multiplication verifications as correct or incorrect by pressing a button with their left or right index finger. The response mappings were counterbalanced between subjects. Instructions at the start of the experiment explained how to perform the verification task and instructed participants to respond both fast and accurately. They were also informed about the need to indicate which strategy they used after each response. Each strategy was then explained in the following way: Recognition: you thought the equation was correct because it looked correct or familiar or you thought the equation was incorrect because it looked wrong or unfamiliar, Remember and compare: You remembered the correct answer of the equation and compared it with the presented solution, Calculate and compare: You calculated the correct answer of the equation and compared it with the presented solution, Other: you used another strategy or you don’t know.

In total there were eight blocks of 112 trials resulting in 896 experimental trials. The experiment started with eight practice trials. During a short break after every block the mean response time of the participant appeared on the screen. The experiment lasted about 60 min.

Each trial started with the presentation of a fixation mark ‘!’ for 500 ms, after which the verification problem appeared on the screen until participants responded or until the response deadline of 1500 ms had passed. After a correct response, a green circle was presented for 500 ms while after an erroneous response a red circle appeared. If participants did not answer within the response interval the words ‘TE TRAAG’ (‘too slow’ in Dutch) appeared on the screen for 500 ms. After the feedback the question ‘Welke strategie heb je gebruikt?’ (‘Which strategy did you use?’ in Dutch) and the four possible options ‘(1) Herkenning, (2) Herinner and vergelijk, (3) Bereken and vergelijk, (4) Iets anders’ [‘(1) Recognition, (2) Remember and compare, (3) Calculate and compare, (4) Other’ in Dutch] appeared on the screen. Participants typed in their answer on a numeric keypad. After a blank screen of 500 ms the subsequent trial started.

## Results

Responses exceeding the response deadline (3% of the data) and subsequent trials were discarded. Furthermore, we removed trials in which strategy four (‘other’) was chosen (4% of the data) as well as the following trial. The first trial after every break was also discarded. As reaction time for strategy choice was also registered, trials on which a decision was made very slow (>2.5SD, 3% of the data) were also removed. In total, 15% of the data was excluded. Two participants were removed from the analyses because they both used only one strategy during the task. For the remaining 18 participants, the mean response time was 886 ms (*SD* = 90 ms). The mean accuracy rate was 81% (*SD* = 9%). On average, participants had at least 55 trials in each cell (*SD* = 35).

Using paired sampled *t*-tests we tested whether strategies differed in proportion of use, accuracy and reaction times. Indeed strategies differed significantly in percentage of use, all *p* ≤ 0.001, and reaction times, all *p* ≤ 0.05. Accuracy differed significantly between recognition and the other two strategies, both *p* ≤ 0.01. The mean accuracy for Remember and Compare and Calculate and Compare did not differ significantly, *t*(16) = -1.34, *p* = 0.20. See **Table 1** for an overview of proportion of use, accuracy and response time for each strategy.

**Table 1:** Mean and standard deviation (between brackets) of proportion of use, accuracy, and response time for each strategy.

Strategy	Proportion of use (%)	Accuracy (%)	Response time (ms)
Recognition	67 (14)	88 (8)	856 (99)
Remember and compare	26 (15)	63 (24)	947 (104)
Calculate and compare	7 (8)	70 (20)	1006 (147)

For the analyses reported below, we applied a linear mixed effects model as implemented in the R-package lme4 (Bates et al., 2013) with a random effect for subjects and strategy. Including a random effect for strategy was done to remove a possible confound as strategies differed significantly in average reaction time, accuracy and proportion of use (see **Table 1**). Consequently, if participants made an error when using a difficult strategy, a subsequent switch to a faster, more efficient strategy (in this case recognition), could influence measures of PES and accuracy. Accuracy and strategy-switch was analyzed using a logistic link function. Both the correct RT data and the proportion of errors were analyzed using the fixed variables previous accuracy and strategy-switch. For the proportion strategy-switch we compared the proportion of switching after a correct response and after an incorrect response. Additionally each variable (i.e., previous accuracy and strategy-switch) was added to the base model as a random slope, for subject and strategy separately, and tested to see if this addition improved the model. If multiple random slopes significantly improved the model, the combination of these variables were added and tested against the models in which the slopes were added separately. As such we acquired a model for reaction time, accuracy and strategy-switch.

### Response Times

The random effect structure for the model for reaction time consisted of both variables, previous accuracy and strategy-switch, as a random slope for subject and strategy-switch as a random slope for strategy.

**FIGURE 1:**
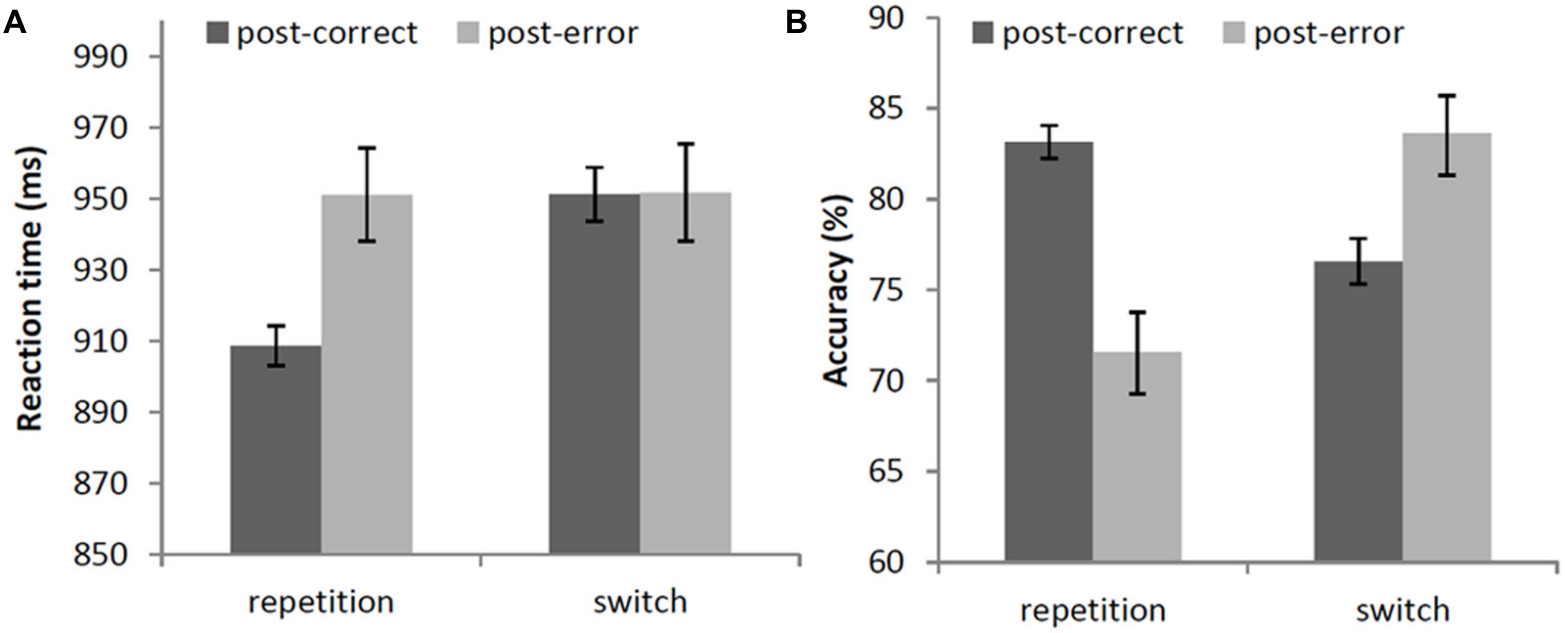
**(A)** Mean response times (in ms) and **(B)** mean accuracy (in percentages) after correct and error trials, depending on a repetition or switch in strategy. Error bars represent 95% confidence intervals around the means.

The main effect of accuracy of the previous trial was significant, χ^2^(1) = 5.90, *p* < 0.05, showing slower responses following an error (952 ms) than following a correct response (930 ms). The main effect of strategy-switch did not reach significance, χ^2^(1) < 0.01, *p* = 0.96, but there was a significant interaction of previous accuracy and strategy-switch, χ^2^(1) = 15.84, *p* < 0.001. When there was a repetition in strategy, response times following errors (951 ms) were significantly slower than trials following a correct response (909 ms), χ^2^(1) = 18.56, *p* < 0.001, resulting in PES (42 ms). When there was a switch in strategy, however, response times following errors (952 ms) were not significantly different from trials following a correct response (951 ms), χ^2^(1) < 0.01, *p* = 0.95. This interaction is shown in **Figure 1A**.

### Accuracy

The random effect structure for the model for accuracy consisted of both variables, previous accuracy and strategy-switch, and their interaction as a random slope for subject and only strategy-switch as a random slope for strategy.

There was a main effect of accuracy of the previous trial, χ^2^(1) = 20.70, *p* < 0.001, showing that participants were less correct following an error (72%) than following a correct response (83%). There was also a main effect of strategy-switch, resp. χ^2^(1) = 15.96, *p* < 0.001. Participants were less correct when they repeated their strategy (72%) than when they switched strategy (84%). Interestingly, the interaction between accuracy of the previous trial and strategy-switch was significant, χ^2^(1) = 11.52, *p* < 0.001. When repeating a strategy, participants were less accurate after an error (72%) than after a correct response (83%), resulting in significant post-error accuracy *decrease* (-11%), χ^2^(1) = 20.70, *p* < 0.001. However, when they switched strategy, participants were more accurate after an error (84%) than after a correct response (77%), resulting in significant post-error accuracy *increase* (+7%), χ^2^(1) = 4.01, *p* < 0.05. See **Figure 1B**.

### Proportion Strategy-Switch

Overall, participants tended to repeat the previous strategy, as mean percentage of strategy switch was only 38% (*SD* = 14%). However, since percentage of use differed significantly between strategies, strategy changes would always be much lower than 66%. We therefore randomized the trials per participant and then re-calculated the percentage of (randomized) strategy switch as a baseline of strategy-switch. Indeed, using a paired samples *t*-test, participants switched less in the experiment than could be expected based on the randomized average strategy switch (*M* = 44%, *SD* = 10%), *t*(17) = -3.86, *p* = 0.001.

To further investigate whether strategy-switch differed depending on previous accuracy we again used a linear mixed effects model with a random effect for subjects and strategy. The random effect structure consisted of a random slope of previous accuracy for both subject and strategy. Interestingly, there was no significant effect of previous accuracy on the amount of strategy switches, χ^2^(1) = 0.15, *p* = 0.70.

### Measuring Post-Error Changes

When investigating post-error adjustments it is important to note that global performance shifts can influence post-error measurements. Dutilh et al. (2012) showed that quantifying PES by subtracting only post-correct trials preceding an error from post-error trials, resulted in a more robust measure of PES. It is possible that participants in our experiment switched strategies more often in the beginning where overall performance was still low, thereby influencing our results. However, when dividing the data into four blocks, no significant difference in percentage of strategy-switch (*p* = 0.56, resp. 48, 54, 52, and 43%) or error rates (*p* = 0.64, resp. 78, 78, 80, and 80%) was found. As such, it is unlikely that global performance shifts influenced our results. Additionally, and in line with these findings, selecting our data based on the method proposed by Dutilh et al. (2012) did not alter the significance of our results.

### Confound of Strategy

Based on significant differences in reaction time, accuracy, and frequency of use of strategies, we included this variable in the random effects structure. When analyzing the data using traditional repeated measures ANOVA’s, or LME models without strategy in the random effect structure, we again found a significant interaction of previous accuracy and strategy switch for both accuracy and reaction times (both *p* ≤ 0.001). Similar to the results reported above, we found significant PES and post-error accuracy decrease when participants repeated their strategy (resp. 32 ms and -12%, both *p* < 0.001). However, in line with our assumption that these differences between strategies might influence our results, the pattern following a strategy switch seemed to be inflated with significant post-error *speeding* and larger post-error accuracy increase (resp. -71 ms and +14%, both *p* ≤ 0.02). Additionally, in these analyses the percentage of strategy-switch also differed significantly following an error (45%) and following a correct response (37%), *F*(1,17) = 6.12, *p* = 0.02.

## Discussion

Following up on the idea that tasks with more degrees of freedom can offer more insight into error monitoring, we investigated strategy-use and performance related to errors in a multiplication verification task. Our data revealed that when participants repeated their strategy there was PES, replicating the typical pattern in studies where only one strategy is possible. But when participants chose another strategy, this PES disappeared. Second and in line with our hypothesis, there is post-error accuracy *increase* when there was a strategy-switch, while repeating the same strategy resulted in post-error accuracy *decrease*. In combination, these results indicate that when participants use a different strategy after an error, this has a positive impact on performance.

While cognitive control theories, such as conflict monitoring theory (Botvinick et al., 2001), predict both PES and increased performance following an error, our results show a different pattern. For strategy repetitions, PES and post-error performance *decrease* is observed, which reflects general decreased performance after errors. This pattern is more in line with non-functional accounts such as the orienting account (Notebaert et al., 2009). However, we used a rather long intertrial interval (at least 1000 ms) and participants had to indicate their strategy between trials. It is therefore unrealistic to assume that in our design an orienting response to an error still influenced post-error performance. Another non-functional account, the bottleneck account (Jentzsch and Dudschig, 2009), predicts decreased performance following errors because of a time and resource consuming error monitoring process. However, this interference is also limited in time (Jentzsch and Dudschig, 2009; Danielmeier and Ullsperger, 2011; Van der Borght et al., 2015). Hence, although the data for strategy repetitions are in line with non-functional accounts for PES, the timing of the experiment makes it unlikely that the specific mechanisms described by these non-functional accounts are at play. Although the precise reason for the performance drop is not clear, it is clear that repeating a strategy that led to an error will further decrease your performance. When participants change strategy, the data pattern alters in two important ways. First, the reaction time is not slower compared to reaction times after correct trials and second, accuracy is higher than after correct trials. Although this is not a typical ‘adaptive’ pattern, it does demonstrate improved performance.

Our pattern of results indicates that strategy changes lead to improved post-error performance. We do need to point out that we did not manipulate strategy selection but rather asked participants to indicate which strategy they used. As such it is possible that third variables, such as attention fluctuation, influence both the likelihood for strategy change and post-error performance. It might therefore be interesting to investigate if similar results are found when participants are instructed to use a specific strategy. Additionally, one could even conceive the (in our opinion unlikely) possibility that participants use (post-error) performance characteristics to indicate which strategy they used.

Additionally, in line with previous findings there is a perseveration effect in strategy choice (Lemaire and Lecacheur, 2010; Schillemans et al., 2011). This resembles the tendency to repeat the same task in voluntary task-switching (Arrington and Logan, 2004). Interestingly, the amount of strategy switches and repetitions did not differ significantly after errors and correct responses. This result indicates that even when an applied strategy led to an erroneous outcome, participants prefer to stay with this strategy, at the cost of reduced performance.

## Conclusion

This experiment shows that the use of commonly used laboratory tasks in research has provided a narrow window on post-error behavior. By using a task where participants had the possibility to change strategies, we demonstrated that participants can indeed increase performance after errors. This increase was observed both in RTs and error rates, contrary to previous reports of a speed-accuracy trade-off following errors (Marco-Pallarés et al., 2008; Danielmeier et al., 2011; Seifert et al., 2011). This is in line with our previous findings and indicates that research on error monitoring should study error monitoring (also) in tasks where participants have cognitive flexibility for real adaptation.

## Author Contributions

LVDB and CD designed the experiment based on previous results and theories of CD and WN. LVDB conducted the experiment, analyzed the data, and provided a first draft of the manuscript. CD and WN further modified the manuscript.

## Conflict of Interest Statement

The authors declare that the research was conducted in the absence of any commercial or financial relationships that could be construed as a potential conflict of interest. The reviewer, Martin Ernst Maier, and handling Editor declared their shared affiliation, and the handling Editor states that the process nevertheless met the standards of a fair and objective review.
